# Analysing the Structural Effect of Point Mutations of Cytotoxic Necrotizing Factor 1 (CNF1) on Lu/BCAM Adhesion Glycoprotein Association

**DOI:** 10.3390/toxins10030122

**Published:** 2018-03-13

**Authors:** Alexandre G. de Brevern

**Affiliations:** Univ Paris Diderot, Sorbonne Paris Cite, Univ de la Réunion, Univ des Antilles, Inserm UMR_S 1134, INTS, Laboratoire d’Excellence GR-Ex, 75015 Paris, France; alexandre.debrevern@univ-paris-diderot.fr; Tel.: +33-144-493-000

**Keywords:** Lu/BCAM, CNF, laminin, toxin, receptor, immunoglobulin-like domain, in silico approaches, computational biology, side-chains, protein-protein interaction, sickle cell disease

## Abstract

Cytotoxic Necrotizing Factor 1 (CNF1) was identified in 1983 as a protein toxin produced by certain pathogenic strains of *Escherichia coli*. Since then, numerous studies have investigated its particularities. For instance, it is associated with the single chain AB-toxin family, and can be divided into different functional and structural domains, e.g., catalytic and transmembrane domain and interaction sites. A few years ago, the identification of the Lutheran (Lu) adhesion glycoprotein/basal cell adhesion molecule (BCAM) as a cellular receptor for CNF1 provided new insights into the adhesion process of CNF1. Very recently, the Ig-like domain 2 of Lu/BCAM was confirmed as the main interaction site using protein-protein interaction and competition studies with various different mutants. Here, I present in silico approaches that precisely explain the impact of these mutations, leading to a better explanation of these experimental studies. These results can be used in the development of future antitoxin strategies.

In 1983 and 1984, Caprioli and coworkers described and characterized for the first time Cytotoxic Necrotizing Factor 1 (CNF1) as a toxin capable of causing multinucleation in cultured cells [1] as well as necrosis in rabbit skin [2]. CNTF1 has been widely analyzed as it is implicated in a large number of uncomplicated urinary tract infections [3]. This 115-kDa cytoplasmic protein is a member of a family of AB-toxins that targets small GTPases [4]. Figure 1 summarizes the topology of the protein, which begins with a N-terminal receptor-binding domain, followed by an amino-acidic region consisting of two short membrane spanning helices, H1 (350–372) and H2 (387–412). This latter region is involved in membrane translocation [5]. The final C-terminal catalytic domain modifies a specific cellular target in the host cell cytosol [6,7]. A few years ago, the identification of the Lutheran (Lu) adhesion glycoprotein/basal cell adhesion molecule (BCAM) as a cellular receptor for CNF1 provided new insights into the adhesion process of CNF1 [8]. Very recently, the Ig-like domain 2 of Lu/BCAM was confirmed as the main interaction site using protein-protein interaction and competition studies with various different mutants [9]. The structure of the catalytic domain of CNTF1 has been available since 2001, yet lacks the region of interest (before position 720). Nonetheless, it is possible to complete it using a threading approach and in silico mutations to precisely explain the results of such experimental mutations.

The structure of the catalytic domain of CNF1 is available as PDB ID 1HQ0 [6]. It corresponds to the positions 720 to 1014. When no structure is available, various different approaches are accessible to tackle this, namely comparative modeling, threading, and de novo approaches [10]. Using PSI-BLAST [11] and ORION [12,13], a good structural template covering the region of positions 657–734 was identified. The corresponding part of the polygalacturonase inhibiting protein (PDB ID 1OGQ [14]), positions 100 to 174 shared slightly higher than 50% similarity with excellent coverage, i.e., nearly no gaps. The prediction of the secondary structure using PSI-PRED correlates very well, emphasizing the quality of the selection of this template to complete CNF1 catalytic domain. A multiple sequence alignment of the different protein chains was generated by ClustalOmega [15] and used with Modeller [16] to build the structural models. One hundred structural models were generated and the best DOPE score was selected and analyzed using classical approaches [17,18].

The different mutations were performed with PyMOL 1.7.1 software [19] using the SCWRL 4.0 tool [20] for (i) simple mutant S720N, (ii) double mutants S720N and S723E, and (iii) double mutants K726S and S727I. Amino acid accessibilities were computed using the DSSP 2.2.1 tool [21] and VLDP webserver [22]. Figure 2 shows different visualizations of the wild-type structural models and mutants, while Table 1 summarizes the change in the accessibility of the residues in this region (additional visualizations can be seen in Supplementary Figures S1–S4).

A striking first result concerns the wild-type (i.e., reference sequence). The addition of the translocation domain made it so that residues 720 and 721 are no longer associated with the helical structures (see Figure 2A and Figure S1). Hence, the helicity of these residues was enhanced by a crystallization process and do not represent the physiological/biological conformation of CNF1.

Reppin and coworkers tested three sets of mutants, beginning with simple mutation S720N, then double mutant S720N and S723E, and finally double mutant K726S and S727I. As seen in Figure 2 of Reference [9], the effects increase from the simple mutant to the last double mutants.

The mutation of residue 720 had no significant difference from S720 to N720 in terms of electrostatics (as seen in Figure 2C,D and Figure S2). It provides a slightly larger accessible surface area for residues 720 and 721 (+20 Å^2^), but strikingly hides the residue 723, i.e., its accessible surface drops from 103 to 8 Å^2^.

The additional change from S723 to E723 does not impact the accessibility of other positions, but it causes the totally inverse action of S720N, providing a larger accessible surface (50% more than WT, see Figure S3). Hence, the combination of these two series of experiments (S720N, then double mutant S720N and S723E) can be confused as (i) a large diminution of the accessibility by S720N of S723, but that is (ii) totally reversed by E723. It thus underlines that the space now taken by E723 provides trouble for the binding of CNF1 mutants to Lu/BCAM.

The last double mutant tested (K726S and S727I) had the strongest impact with a total change in the local charge, causing the surface to become more flat and negative with huge change in the accessibility area (see Figure 1E and Figure S4).

The analyses at the view of in silico structural models underline various different interesting facts. Firstly, all of these results underline that whatever the local conformation around residue 720, it seems to have no real impact in the binding of CNF1 to Lu/BCAM.

Secondly, in contrast, the positions 723, 726, and 727 seem to have potential roles in the binding. However, it is slightly more complex than a simple direct binding effect. Indeed, position 723 (with respect to simple mutant S720N) had a slight but steric effect on the binding.

Finally, the last double mutant, K726S and S727I, is so stringent that both accessibility and electrostatics are scrambled. The only problematic issue is that it would have been more beneficial to have independent mutants for each of them. As position 726 was the most accessible and the change of charge is important, it might be the driving force behind the change in the binding of CNF1 to Lu/BCAM.

To conclude, these different analyses underline the importance (i) of having the environment (when possible) to precisely analyze the effect of mutants/variants and (ii) of using 3D information to determine the effects of such mutations, as some effects are not so obvious or easily analyzed.

Using such methodology with CNF1 binding to Lu/BCAM, it would be possible to design mutants with expected effects and test them via in vitro experiments. These results can be used in the development of future antitoxin strategies.

## Figures and Tables

**Figure 1:**
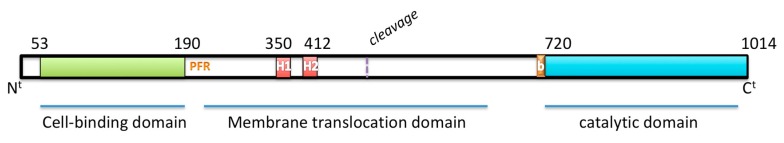
Topology of Cytotoxic Necrotizing Factor 1 CNF1. Shown are the cell-binding domain, the membrane translocation domain with its two (putative) helices, and the catalytic domain. Also shown are the Pore Forming Region (PFR) and the cleavage position, while (b) represents the positions that were studied in Reference [9] and are not present in the crystal structure.

**Figure 2:**
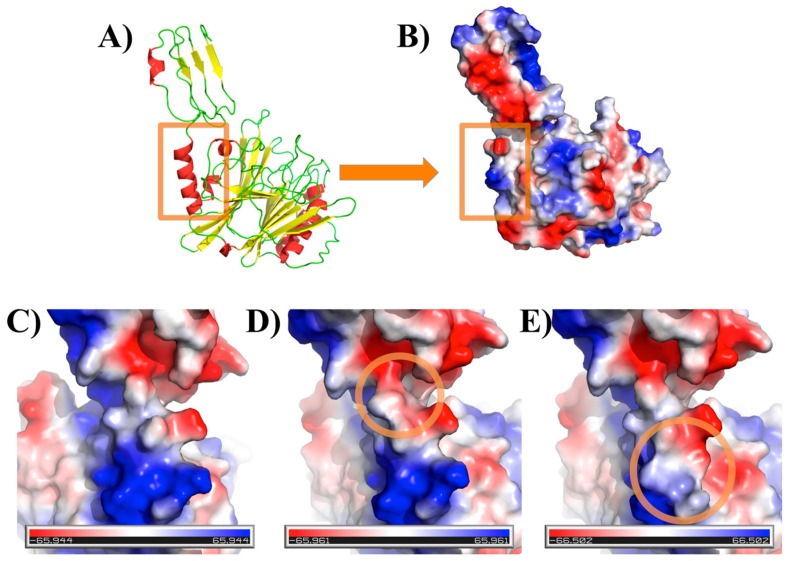
Structural models of 657–1014 CNF1 fragment. (**A**) The structural model of the wild-type in cartoon representation and (**B**) with electrostatic representation is shown. The orange square represents the interaction zone observed in this study. (**C**–**E**) are a focus on this region with electrostatic representation with (**C**) being the wild-type, (**D**) the S720N, and (**E**) the K726S and S727I regions, highlighted by an orange circle. All visualizations were created with PyMOL software [19].

**Table 1:** Accessibility of CNF1 for positions 720 to 727 using DSSP [21] for the wild-type and three series of mutants. VLDP [22] provides equivalent results.

	Accessibility			
Residue Number	Wild-Type	S720N	S720N & S723E	K726S & S727I
720	100	122	120	100
721	2	23	23	23
722	165	162	165	157
723	103	8	153	95
724	48	48	45	46
725	4	4	4	3
726	138	138	138	61
727	38	38	38	80

## Supplementary Materials

The following are available online at http://www.mdpi.com/2072-6651/10/3/122/s1, Figure S1: Structural model of wild-type CNF1 657–1024 domain; Figure S2: Structural model of mutant S720N of CNF1 657–1024 domain; Figure S3: Structural model of double mutant S720N and S723E of CNF1 657–1024 domain; Figure S4: Structural model of double mutant K726S and S727I of CNF1 657–1024 domain.
