# Supplementary material for: Analysing the Structural Effect of Point Mutations of Cytotoxic Necrotizing Factor 1 (CNF1) on Lu/BCAM Adhesion Glycoprotein Association
Source: Toxins (Basel). 2018 Mar 13;10(3):122. doi: 10.3390/toxins10030122 (PMC5869410; doi:10.3390/toxins10030122)
Supplement: Supplementary file 1 [file toxins-10-00122-s001.docx]

Analyzing the structural effect of point mutations of Cytotoxic Necrotizing Factor 1 (CNF1) to Lu/BCAM Adhesion Glycoprotein association

Alexandre G. de Brevern


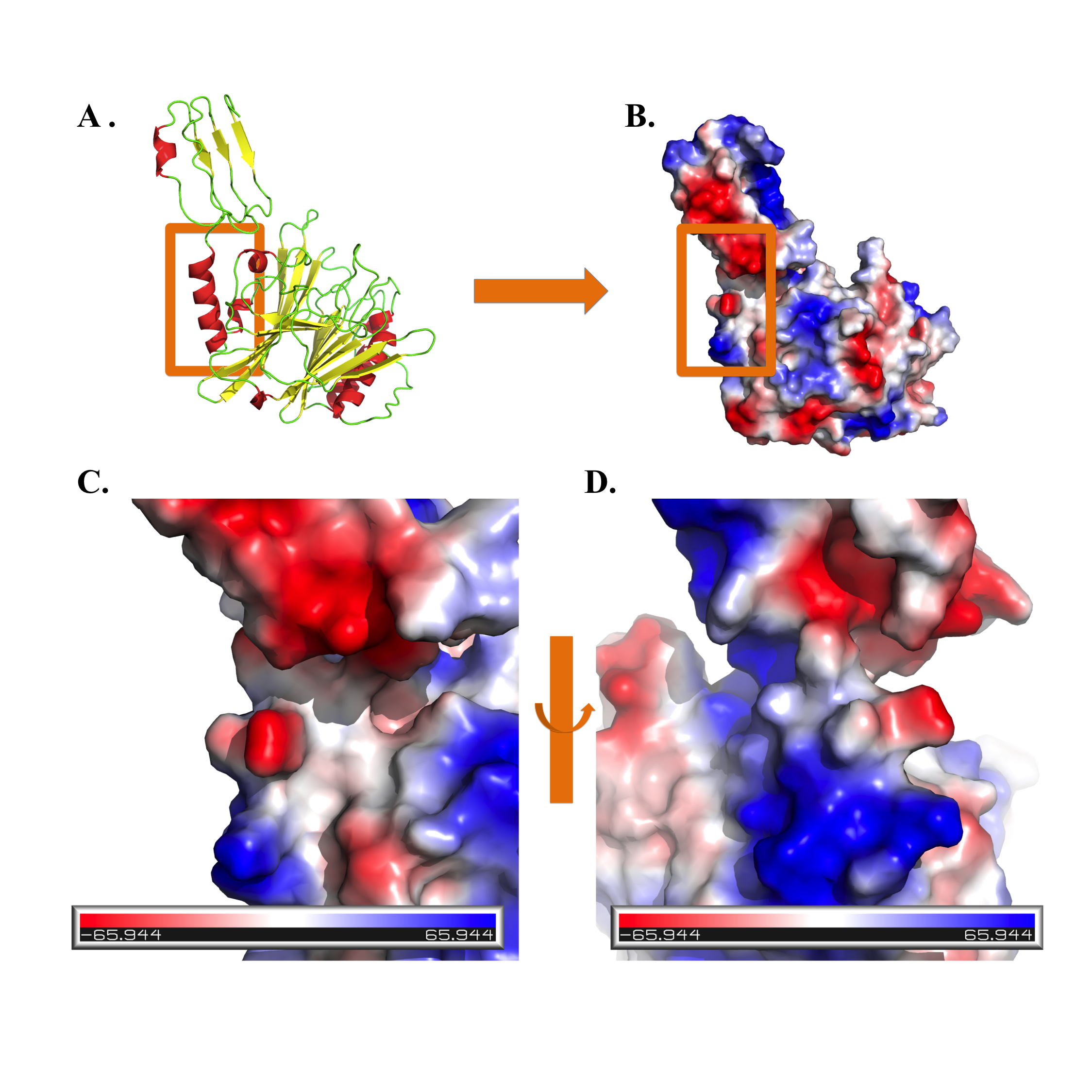


**Figure S1**. Structural model of wild type CNF1 657-1024 domain. (**A**). Cartoon representation. (**B**). Electrostatics visualisation. (**C**) & (**D**). Focus on the interaction region.


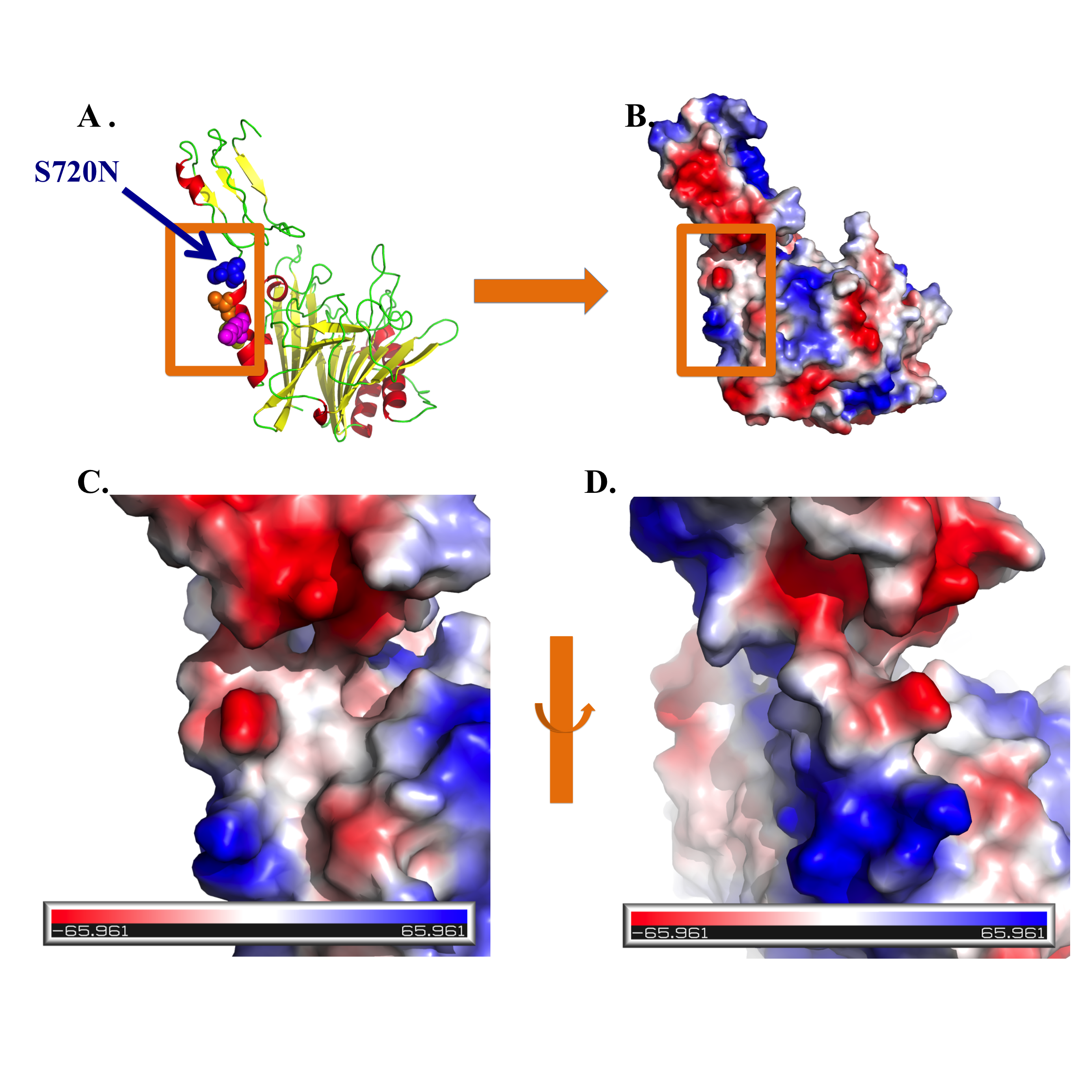


**Figure S2**. Structural model of mutant S720N of CNF1 657-1024 domain. (**A**). Cartoon representation. (**B**). Electrostatics visualisation. (**C**) & (**D**). Focus on the interaction region.


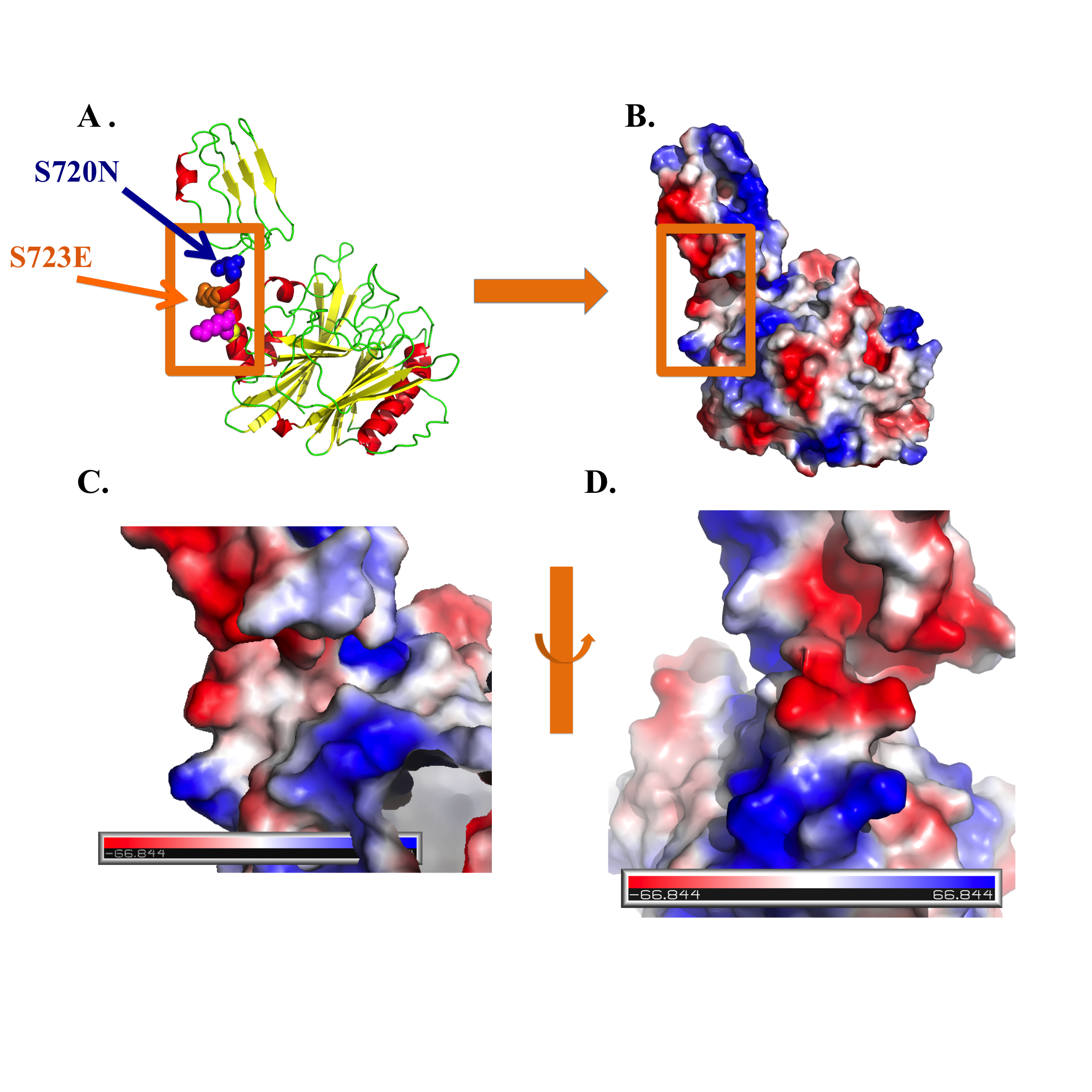


**Figure S3**. Structural model of double mutant S720N & S723E of CNF1 657-1024 domain. (**A**). Cartoon representation. (**B**). Electrostatics visualisation. (**C**) & (**D**). Focus on the interaction region.


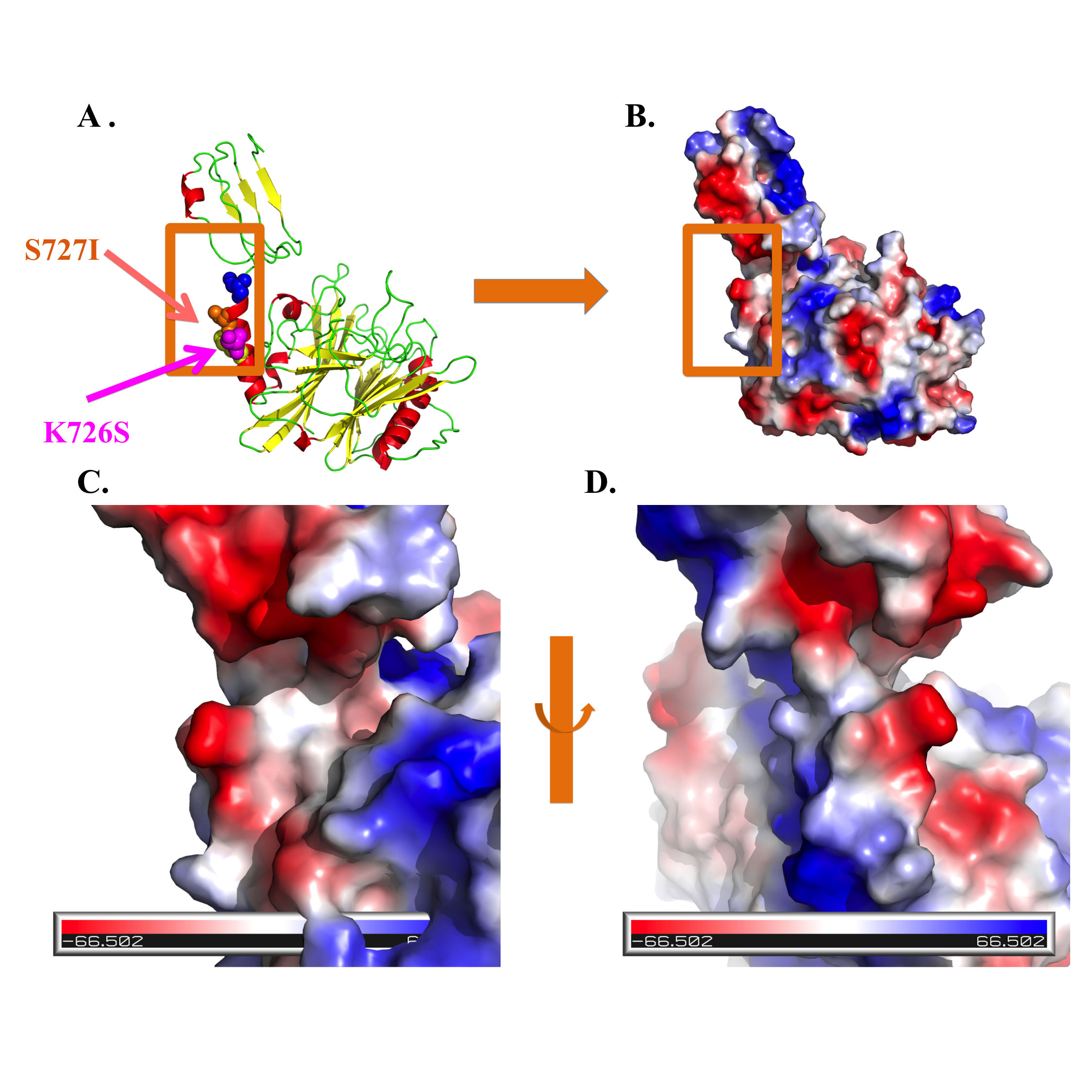


**Figure S4**. Structural model of double mutant K726S & S727I of CNF1 657-1024 domain. (**A**). Cartoon representation. (**B**). Electrostatics visualisation. (**C**) & (**D**). Focus on the interaction region.
